# Causal relationship between vitamin D and adult height: A bidirectional Mendelian randomization study

**DOI:** 10.1097/MD.0000000000044123

**Published:** 2025-08-29

**Authors:** Lianhui Chen, Min Wu, Zhenzhong Zeng, Xiaohao Hu, Yongfen Wang

**Affiliations:** a Department of Pediatrics, The Second Affiliated Hospital of Fujian Medical University, Quanzhou, China; b Rare Disease Medical Center, The Second Affiliated Hospital of Fujian Medical University, Quanzhou, China.

**Keywords:** 25-hydroxyvitamin D, adult height, linear growth, Mendelian randomization, vitamin D

## Abstract

Vitamin D is critical for skeletal growth, but its causal link to adult height remains uncertain due to limitations in observational studies and randomized controlled trials. This study aimed to explore the bidirectional causal relationship between circulating vitamin D levels and adult height using Mendelian randomization (MR). A bidirectional 2-sample MR analysis was performed with large-scale genome-wide association study data. Vitamin D data were sourced from the Pan-UKB (n = 383,324 European individuals), and height data from FinnGen R12 (n = 364,629 Finnish individuals). Genetic instruments were selected based on genome-wide significance (*P* < 5 × 10^‐8^) and independence (linkage disequilibrium *r*² < 0.001). Multiple MR approaches, including inverse variance weighted (IVW), MR-Egger, and weighted median, were applied, with sensitivity analyses to evaluate pleiotropy and heterogeneity. Genetically predicted higher vitamin D concentrations were associated with increased adult height, with each standard deviation increase in vitamin D corresponding to a 0.046 standard deviation height increase (*P* = 1.53 × 10^‐5^, IVW method). Sensitivity analyses supported this finding, showing no directional pleiotropy. Conversely, no causal effect of height on vitamin D levels was detected (β = 0.008, *P* = .343, IVW method). This study provides genetic evidence supporting a modest causal effect of circulating vitamin D levels on adult height, with no evidence of reverse causality. These findings complement and extend prior randomized controlled trials evidence, highlighting the role of vitamin D in skeletal development while underscoring that supplementation alone is unlikely to yield substantial height gains in vitamin D-replete populations.

## 1. Introduction

Vitamin D is an essential fat-soluble nutrient primarily known for its critical role in regulating calcium and phosphate homeostasis, thereby contributing significantly to skeletal growth and maintenance.^[1]^ Genetic studies have shown that polymorphisms in the vitamin D receptor (VDR) gene are associated with variations in human height and may also influence the growth response to recombinant human growth hormone therapy in children with idiopathic short stature.^[2–4]^ Observational evidence indicates that inadequate vitamin D levels during critical growth periods, particularly childhood and adolescence, may impair linear growth, potentially leading to compromised adult height.^[5,6]^

However, observational studies are vulnerable to confounding and reverse causation, making it difficult to establish causality. Recent large-scale randomized controlled trials (RCTs) have also provided mixed findings: while supplementation effectively increases circulating 25-hydroxyvitamin D (25OHD) levels, it does not consistently promote linear growth or improve body composition in children and adolescents.^[7–11]^ Trials conducted in Mongolia,^[9]^ South Africa,^[12]^ Afghanistan,^[8]^ and comprehensive meta-analyses^[13–15]^ all suggest that vitamin D supplementation alone may have limited effects on growth outcomes during childhood. These results highlight the complexity of the relationship and suggest that factors such as timing of intervention, baseline nutritional status, and coexisting deficiencies may modify the effects of vitamin D on growth.

To address the limitations inherent in observational studies and overcome the short duration of most RCTs, Mendelian randomization (MR) offers a robust alternative by using genetic variants as proxies for exposures.^[16]^ Because genetic variants are randomly allocated at conception, MR analysis can minimize confounding and reverse causality, providing more reliable estimates of long-term causal effects.

In this study, we conducted a bidirectional 2-sample MR analysis using large-scale genome-wide association study (GWAS) datasets to investigate the potential causal relationship between circulating vitamin D concentrations and adult height. Our objectives were to: clarify whether genetically predicted vitamin D concentrations influence adult height; and determine if genetically predicted adult height impacts circulating vitamin D levels. By applying this robust analytical framework, we aimed to provide conclusive evidence that could inform clinical guidelines and public health strategies for optimizing growth and nutritional interventions.

## 2. Methods

### 2.1. Study design

**Figure 1.**
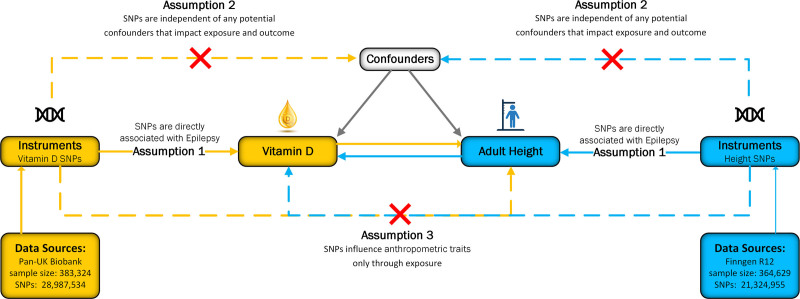
Study overview and Mendelian randomization model. Schematic diagram illustrating the bidirectional MR study investigating the causal relationship between circulating vitamin D concentrations and adult height. The figure highlights the key assumptions underpinning MR analysis: (1) genetic variants must be strongly associated with the exposure (vitamin D levels or height); (2) genetic variants must be independent of confounding factors; and (3) genetic variants must influence the outcome solely through the exposure, without alternative pathways.

This study employed a bidirectional, 2-sample MR design, leveraging large-scale GWAS datasets to test potential causal effects from circulating vitamin D concentrations to adult height and vice-versa. As illustrated in Figure 1, MR inference rests on 3 core instrumental-variable assumptions: relevance, independence, and exclusion restriction, which together ensure that the genetic instruments approximate randomized allocation of the exposure.^[17]^ The design, conduct, and reporting of this study adhered to the strengthening the reporting of observational studies in epidemiology using Mendelian randomization checklist, and a completed checklist is provided in the Supplementary Materials S1, Supplemental Digital Content, http://links.lww.com/MD/P885.^[18,19]^ This study utilized publicly available summary statistics from GWAS. All underlying studies received ethical approval from their respective institutional review boards, and participants provided informed consent. No individual-level data were used, and no new ethical approval was necessary.

### 2.2. Data sources

Due to the bidirectional nature of this analysis, each trait served once as the exposure and once as the outcome. As shown in Table S1, Supplemental Digital Content, https://links.lww.com/MD/P766, for vitamin D (25OHD), we used GWAS data from the Pan-UKB (http://www.nealelab.is/uk-biobank/), which recruited approximately 500,000 participants aged 40 to 69 years from across the UK between 2006 and 2010.^[20]^ For this study, only individuals of European ancestry were included (n = 383,324, SNPs = 28,987,534). Vitamin D levels were measured using the DiaSorin Liaison chemiluminescent immunoassay, which employs a direct competitive method to quantify vitamin D in serum, and extreme values outside the assay range (10–375 nmol/L) were excluded. The average within-laboratory coefficient of variation ranged from 5.04% to 6.14%. To maintain the integrity of the measurements, regular external quality assurance assessments were conducted, with the vitamin D assay achieving a 100% acceptable performance rate. GWAS analysis for vitamin D was conducted using the linear mixed model, adjusting for age, sex, month of assessment, center, supplement intake, genotyping batch, and the first 40 genetic principal components.

Adult height data were derived from FinnGen R12, which includes 500,348 individuals of Finnish ancestry.^[21]^ Among them, 364,629 had available height data (inverse-rank-normal transformed) and 21,324,955 SNPs were included. GWAS for height was performed using the regenie with adjustment for age, sex, 10 principal components, and genotyping batch.

### 2.3. Instrumental-variable selection

Instrumental variables for vitamin D and height were selected based on genome-wide significance (*P* < 5 × 10^‐8^), independence (linkage disequilibrium [LD] *r*² < 0.001), and clumping distance of 10 Mb. A total of 82 independent SNPs associated with vitamin D concentrations and 705 independent SNPs associated with height were utilized as instruments prior to harmonization and outlier filtering.

### 2.4. Mendelian randomization analyses

We performed MR analyses using 5 complementary methods: inverse variance weighted (IVW), MR-Egger, weighted median, simple mode, and weighted mode. The IVW method served as the primary analytical approach, providing the most efficient estimation under valid instrument assumptions.^[22]^ The estimators from other 4 methods were included as robust sensitivity analyses. All effect estimates are reported as β coefficients representing the standard deviation (SD) change in the outcome per SD increase in the exposure.

### 2.5. Sensitivity analysis

We employed the MR-PRESSO test to detect and exclude outlying SNPs that could disproportionately influence the causal estimate.^[23,24]^ When the outlier test indicated significant heterogeneity (*P* < .05), outlier variants were removed and the estimates was recalculated on the cleaned instrument set. The validity of the MR assumptions was assessed through heterogeneity tests (Cochran *Q* and *I*^2^ statistics) and the MR-Egger intercept test for horizontal pleiotropy.^[25,26]^ Directionality and causal inference were further evaluated using the Steiger test to confirm the validity of the causal direction from exposure to outcome. Leave-one-out sensitivity analyses were also performed to evaluate the robustness of the results.

### 2.6. Statistical analysis

All MR analyses were performed using the TwoSampleMR package (version 0.6.2) in R software (version 4.4.0; R Foundation for Statistical Computing, Vienna, Austria).^[16]^ Statistical significance was determined at a *P*-value of <.05. Multiple testing across bidirectional hypotheses (2 primary tests) was controlled using Bonferroni correction (α = 0.025).

## 3. Results

### 3.1. Instrument strength and SNP selection

We identified 82 genome-wide significant and independent SNPs for vitamin D and 705 for height based on standard clumping criteria. After harmonization with outcome data and removal of outliers using the MR-PRESSO method, 65 SNPs remained in the vitamin D on height direction and 669 SNPs in the height on vitamin D direction. The strength of these instrumental variables was confirmed by F-statistics ranging from 29.8 to 1816.0 for vitamin D and from 29.8 to 948.4 for height, well above the conventional threshold of 10, minimizing the risk of weak instrument bias. A complete list of the SNPs used in the final analyses is provided in Table S2, Supplemental Digital Content, https://links.lww.com/MD/P767.

### 3.2. Causal effect of vitamin D on height

**Figure 2.**
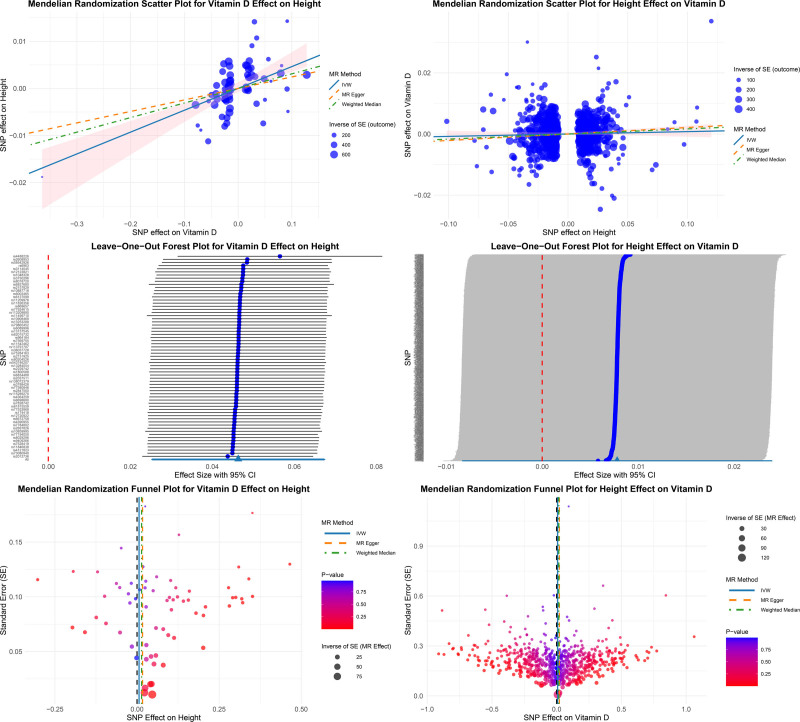
Scatter, leave-one-out, and funnel plots for bidirectional Mendelian randomization analyses. Summary plots displaying the causal estimates from bidirectional MR analyses between vitamin D concentrations and adult height. Effect estimates are shown with 95% confidence intervals.

The MR analysis indicated a significant positive causal effect of vitamin D concentrations on adult height. As shown in Table 1 and Figure 2, the primary IVW method estimated that each 1 SD increase in genetically predicted 25OHD concentration was associated with a 0.046 SD increase in adult height (*P* = 1.53 × 10^‐5^). Supporting this result, both the weighted median (β = 0.031, *P* = 2.74 × 10^‐3^) and weighted mode (β = 0.034, *P* = 2.10 × 10^‐4^) methods yielded consistent effect estimates. While the MR-Egger point estimate was positive, it did not reach statistical significance (*P* = .148), and the MR-Egger intercept test revealed no evidence of directional pleiotropy (*P* = .094). Moderate heterogeneity was observed across instruments (*I*^2^ = 59.5%). The Steiger directionality test confirmed the direction of effect from vitamin D to height (*P* reported as 0 due to computational lower bound).

**Table 1:** Mendelian randomization estimates of the causal effect between vitamin D and height.

Method	No. of SNPs	MR analysis			Heterogeneity Test			*P* of MR-Egger intercept	Steiger test	
		β	SE	*P*	Cochran *Q*	*I* ^2^	*P*		Direction	*P*
Vitamin D on height										
IVW	65	0.046	0.011	1.53E‐05	158.1	59.5	6.33E‐10	–	True	0*
MR-Egger	65	0.024	0.017	1.48E‐01	151.1	58.3	3.32E‐09	.094		
Weighted median	65	0.031	0.010	2.74E‐03	–	–	–	–		
Simple mode	65	0.042	0.021	5.02E‐02	–	–	–	–		
Weighted mode	65	0.034	0.009	2.10E‐04	–	–	–	–		
Height on vitamin D										
IVW	669	0.008	0.008	.343	1117.7	40.2	3.34E‐25	–	True	0*
MR-Egger	669	0.021	0.019	.262	1116.6	40.3	3.18E‐25	.432		
Weighted median	669	0.017	0.012	.160	–	–	–	–		
Simple mode	669	0.023	0.035	.516	–	–	–	–		
Weighted mode	669	0.014	0.029	.633	–	–	–	–		

Height was corrected using inverse-rank normalization.

Vitamin D concentration is expressed in nmol/L.

* *P*-value is reported as 0 due to computational limits.

### 3.3. Causal effect of height on vitamin D

In the reverse direction, we found no evidence to support a causal effect of adult height on circulating vitamin D levels. As shown in Table 1 and Figure 2, the IVW estimate was small and nonsignificant (β = 0.008, *P* = .343). Other MR methods, including MR-Egger (*P* = .262) and weighted median (*P* = .160), were consistent in direction and statistical insignificance. No directional pleiotropy was detected (MR-Egger intercept *P* = .432), and the Steiger test again validated the causal orientation (*P* reported as 0 due to computational lower bound). While there was moderate heterogeneity among instruments (*I*^2^ = 40.2%), the findings across sensitivity analyses converged on the absence of a reverse causal effect.

## 4. Discussion

In this bidirectional 2-sample MR study, we found evidence supporting a causal relationship between genetically predicted circulating vitamin D concentrations and adult height, while no causal effect of genetically predicted height on vitamin D levels was observed. The consistency across multiple MR estimators, together with the absence of substantial horizontal pleiotropy, strengthens the robustness of these findings. Our results highlight that higher 25OHD levels modestly promote linear growth, whereas the associations between taller stature and lower vitamin D levels observed in previous observational studies are likely attributable to confounding or reverse causality rather than a true biological pathway.^[27]^

We chose adult vitamin D concentrations as the exposure because large-scale GWAS datasets for circulating 25OHD during childhood are not yet available. Importantly, the major genetic variants influencing vitamin D metabolism, such as GC and CYP2R1, exert stable effects across the life course, supporting the use of adult-based genetic instruments as proxies for long-term systemic exposure, including during critical growth periods.^[28,29]^ Nevertheless, gene-environment interactions unique to childhood could attenuate the observed effects, possibly contributing to the modest effect size detected.

Our findings are consistent with, yet extend beyond, the evidence from RCTs. Several large-scale RCTs have demonstrated that although vitamin D supplementation significantly raises circulating 25OHD levels, it does not substantially enhance linear growth in children and adolescents.^[8,9,12]^ For example, phase 3 trials conducted in Mongolian (14,000 IU/wk for 3 years) and South African children (10,000 IU/wk for 3 years) failed to show significant growth benefits after long-term supplementation, in contrast to an earlier phase 2 trial in Mongolian children, where daily supplementation with 800 IU of vitamin D_3_ for 6 months led to a modest but statistically significant increase in height among those with very low baseline serum vitamin D levels.^[9,12,30]^ While this pattern seems to suggest that daily vitamin D supplementation may be more effective than weekly regimens in promoting linear growth, no head-to-head trials have directly compared the 2 dosing schedules in terms of height outcomes. However, a randomized controlled trial in Indian children with nutritional rickets did report superior improvements in radiological healing and serum 25OHD levels in the daily supplementation group compared to weekly regimens, supporting the biological plausibility of daily dosing having a stronger skeletal effect.^[31]^ We therefore advocate for future trials to directly compare the impact of daily versus intermittent vitamin D supplementation on linear growth in at-risk pediatric populations.

While such findings highlight the potential influence of dosing strategy, meta-analyses have nonetheless corroborated the overall lack of significant effects of vitamin D supplementation on height.^[13,14]^ However, it is important to recognize that these RCTs may have been underpowered to detect modest effects due to small expected effect sizes, relatively short follow-up durations, and limited sample sizes. By contrast, MR analyses leverage genetic variants as lifelong proxies for exposure, providing greater statistical power to detect small but genuine causal effects. The positive association observed in our study suggests that vitamin D does contribute to linear growth, but the magnitude of its influence is too subtle to be readily captured within the constraints of conventional RCT designs.

From a biological perspective, vitamin D promotes skeletal development by facilitating calcium and phosphate absorption, stimulating chondrocyte proliferation within the growth plate, and modulating insulin-like growth factor-1 signaling.^[1,32,33]^ Severe deficiency leads to rickets and growth failure, as observed in VDR-knockout models and clinical settings.^[34,35]^ Recent 2-sample Mendelian randomization analyses have further supported a bidirectional causal relationship between circulating 25OHD and insulin-like growth factor-1 levels.^[36]^ Notably, 25OHD is the precursor form of vitamin D and requires hydroxylation to 1,25-dihydroxyvitamin D (1,25(OH)_2_D), the biologically active form that binds to the VDR to exert genomic effects.^[37]^ Impaired conversion to 1,25(OH)_2_D, potentially due to genetic polymorphisms in CYP27B1 or other related enzymes, may attenuate the skeletal actions of vitamin D. However, due to the lack of large-scale GWAS for 1,25(OH)_2_D, we were unable to include this active form in our current MR analyses.

Despite these mechanistic insights, our estimates suggest that in vitamin D-replete populations, each 1-SD increase in 25OHD is associated with only a 0.046 SD increase in height, corresponding to a clinically modest impact. From a public health perspective, our results reinforce the importance of maintaining adequate vitamin D status for skeletal health but indicate that supplementation alone is unlikely to produce substantial height gains in otherwise healthy individuals. Therefore, strategies should prioritize preventing overt vitamin D deficiency, particularly during critical periods of growth, rather than expecting notable gains in stature through universal supplementation.

Several strengths enhance the validity of our study, including the large sample sizes of both exposure and outcome GWAS datasets, restriction to European ancestry to minimize population stratification, use of multiple complementary MR methods, and rigorous sensitivity analyses such as MR-PRESSO and Steiger tests. We strictly adhered to the strengthening the reporting of observational studies in epidemiology using Mendelian randomization reporting guidelines, ensuring transparency and reproducibility.

Nonetheless, certain limitations warrant consideration. First, although no substantial pleiotropy was detected, residual pleiotropy cannot be completely excluded. Second, the use of adult vitamin D instruments to proxy developmental exposures may underestimate the true causal effect during specific growth windows. Third, height was inverse-rank normalized in the FinnGen cohort, complicating the translation of effect sizes into centimeters. Finally, our findings may not generalize to non-European populations, where genetic determinants of vitamin D status and environmental exposures differ.

## 5. Conclusion

This bidirectional MR study provides genetic evidence for a modest causal effect of circulating vitamin D concentrations on adult height, with no evidence of reverse causality. Our findings complement existing RCT evidence and underscore the complex role of vitamin D in skeletal development. Further research is needed to refine life stage-specific exposures and validate these results across diverse populations.

## Acknowledgments

The authors would like to acknowledge the Pan-UK Biobank project for providing the GWAS summary statistics on circulating vitamin D concentrations, and the FinnGen consortium for contributing the GWAS data on adult height. We also extend our gratitude to all the researchers and participants involved in these consortia, and to the broader scientific community for making GWAS summary-level data publicly available, which was instrumental for the completion of this study.

## Author contributions

**Conceptualization:** Lianhui Chen.

**Data curation:** Min Wu, Xiaohao Hu.

**Methodology:** Min Wu, Zhenzhong Zeng.

**Software:** Min Wu, Zhenzhong Zeng.

**Supervision:** Yongfen Wang.

**Validation:** Xiaohao Hu.

**Writing – original draft:** Lianhui Chen.

**Writing – review & editing:** Xiaohao Hu, Yongfen Wang.

## Supplementary Material


