# Supplementary material for: Causal relationship between vitamin D and adult height: A bidirectional Mendelian randomization study
Source: Medicine (Baltimore). 2025 Aug 29;104(35):e44123. doi: 10.1097/MD.0000000000044123 (PMC12401308; doi:10.1097/MD.0000000000044123)
Supplement: Supplementary file 1 [file medi-104-e44123-s001.pdf]

STROBE-MR checklist of recommended items to address in reports of Mendelian randomization studies[1 2]

Item No.	Section	Checklist item	Page No.	Relevant text from manuscript
1	**TITLE and ABSTRACT**	Indicate Mendelian randomization (MR) as the study's design in the title and/or the abstract if that is a main purpose of the study	3	Title: Causal Relationship Between Vitamin D and Adult Height: A Bidirectional Mendelian Randomization Study
	**INTRODUCTION**			
2	**Background**	Explain the scientific background and rationale for the reported study. What is the exposure? Is a potential causal relationship between exposure and outcome plausible? Justify why MR is a helpful method to address the study question	5	Vitamin D is an essential fat-soluble nutrient primarily known for its critical role in regulating calcium and phosphate homeostasis, thereby contributing significantly to skeletal growth and maintenance(1). Genetic studies have shown that polymorphisms in the vitamin D receptor (VDR) gene are associated with variations in human height and may also influence the growth response to recombinant human growth hormone therapy in children with idiopathic short stature(2-4). Observational evidence indicates that inadequate vitamin D levels during critical growth periods, particularly childhood and adolescence, may impair linear growth, potentially leading to compromised adult height (5, 6).<br>However, observational studies are vulnerable to confounding and reverse causation, making it difficult to establish causality. Recent large-scale randomized controlled trials (RCTs) have also provided mixed findings: while supplementation effectively increases circulating 25-hydroxyvitamin D (25OHD) levels, it does not consistently promote linear growth or improve body composition in children and adolescents(7-11). Trials conducted in Mongolia (9), South Africa(12), Afghanistan (8), and comprehensive meta-analyses (13-15) all suggest that vitamin D supplementation alone may have limited effects on growth outcomes during childhood. These results highlight the complexity of the relationship and suggest that factors such as timing of intervention, baseline nutritional status, and coexisting deficiencies may modify vitamin D's effects on growth.

				To address the limitations inherent in observational studies and overcome the short duration of most RCTs, Mendelian randomization (MR) offers a robust alternative by using genetic variants as proxies for exposures(16). Because genetic variants are randomly allocated at conception, MR analysis can minimize confounding and reverse causality, providing more reliable estimates of long-term causal effects.
3	**Objectives**	State specific objectives clearly, including pre-specified causal hypotheses (if any). State that MR is a method that, under specific assumptions, intends to estimate causal effects	6	In this study, we conducted a bidirectional two-sample MR analysis using large-scale genome-wide association study (GWAS) datasets to investigate the potential causal relationship between circulating vitamin D concentrations and adult height. Our objectives were to: (1) clarify whether genetically predicted vitamin D concentrations influence adult height, and (2) determine if genetically predicted adult height impacts circulating vitamin D levels. By applying this robust analytical framework, we aimed to provide conclusive evidence that could inform clinical guidelines and public health strategies for optimizing growth and nutritional interventions.
	**METHODS**			
4	**Study design and data sources**	Present key elements of the study design early in the article. Consider including a table listing sources of data for all phases of the study. For each data source contributing to the analysis, describe the following:		
	a)	Setting: Describe the study design and the underlying population, if possible. Describe the setting, locations, and relevant dates, including periods of recruitment, exposure, follow-up, and data collection, when available.	6-7	This study employed a bidirectional, two-sample MR design, leveraging large-scale GWAS datasets to test potential causal effects from circulating vitamin D concentrations to adult height and vice-versa.
	b)	Participants: Give the eligibility criteria, and the sources and methods of selection of participants. Report the sample size, and whether any power or sample size calculations were carried out prior to the main analysis	7	Due to the bidirectional nature of this analysis, each trait served once as the exposure and once as the outcome. For vitamin D (25-hydroxyvitamin D, 25OHD), we used GWAS data from the Pan-UKB (http://www.nealelab.is/uk-biobank/), which recruited approximately 500,000 participants aged 40–69 years from across the UK between 2006 and 2010(20). For this study, only individuals of European ancestry were included (n = 383,324, SNPs = 28,987,534).

					Adult height data were derived from FinnGen R12, which includes 500,348 individuals of Finnish ancestry(21). Among them, 364,629 had available height data (inverse-rank-normal transformed) and 21,324,955 SNPs were included. GWAS for height was performed using the regenie with adjustment for age, sex, 10 PCs, and genotyping batch.
		c)	Describe measurement, quality control and selection of genetic variants	7-8	Instrumental variables (IVs) for vitamin D and height were selected based on genome-wide significance ($p < 5 \times 10^{-8}$), independence (LD $r^2 < 0.001$), and clumping distance of 10 Mb. A total of 82 independent SNPs associated with vitamin D concentrations and 705 independent SNPs associated with height were utilized as instruments prior to harmonization and outlier filtering.
		d)	For each exposure, outcome, and other relevant variables, describe methods of assessment and diagnostic criteria for diseases	7	Vitamin D levels were measured using the DiaSorin Liaison® chemiluminescent immunoassay, and extreme values outside the assay range (10–375 nmol/L) were excluded. The average within-laboratory coefficient of variation ranged from 5.04% to 6.14%.
		e)	Provide details of ethics committee approval and participant informed consent, if relevant	1	This study utilized publicly available summary statistics from the Pan-UK Biobank and FinnGen R12 genome-wide association studies. All underlying studies received ethical approval from their respective institutional review boards, and participants provided informed consent. No individual-level data were used, and no new ethical approval was necessary. The research complied with all relevant regulations regarding the secondary use of human genetic data.
5	**Assumptions**		Explicitly state the three core IV assumptions for the main analysis (relevance, independence and exclusion restriction) as well assumptions for any additional or sensitivity analysis	6	As illustrated in Figure 1, MR inference rests on three core instrumental-variable assumptions: relevance, independence, and exclusion restriction, which together ensure that the genetic instruments approximate randomized allocation of the exposure(17).
6	**Statistical methods: main analysis**		Describe statistical methods and statistics used		
		a)	Describe how quantitative variables were handled in the analyses (i.e., scale, units, model)	7	Vitamin D levels were measured using the DiaSorin Liaison® chemiluminescent immunoassay, and

					extreme values outside the assay range (10–375 nmol/L) were excluded.
		b)	Describe how genetic variants were handled in the analyses and, if applicable, how their weights were selected	8	We performed MR analyses using five complementary methods: inverse variance weighted (IVW), MR-Egger, weighted median, simple mode, and weighted mode. The IVW method served as the primary analytical approach, providing the most efficient estimation under valid instrument assumptions(22).
		c)	Describe the MR estimator (e.g. two-stage least squares, Wald ratio) and related statistics. Detail the included covariates and, in case of two-sample MR, whether the same covariate set was used for adjustment in the two samples	7	GWAS analysis for vitamin D was conducted using the linear mixed model, adjusting for age, sex, month of assessment, center, supplement intake, genotyping batch, and the first 40 genetic principal components (PC).<br>GWAS for height was performed using the regenie with adjustment for age, sex, 10 PCs, and genotyping batch.
		d)	Explain how missing data were addressed	8	We employed the MR-PRESSO test to detect and exclude outlying SNPs that could disproportionately influence the causal estimate.(23, 24) When the outlier test indicated significant heterogeneity ($p < 0.05$), outlier variants were removed and the estimates was recalculated on the cleaned instrument set.
		e)	If applicable, indicate how multiple testing was addressed	9	Multiple testing across bidirectional hypotheses (two primary tests) was controlled using Bonferroni correction ($\alpha = 0.025$).
7	**Assessment of assumptions**		Describe any methods or prior knowledge used to assess the assumptions or justify their validity		The validity of the MR assumptions was assessed through heterogeneity tests (Cochran's Q and I^2 statistics) and the MR-Egger intercept test for horizontal pleiotropy(25, 26). Directionality and causal inference were further evaluated using the Steiger test to confirm the validity of the causal direction from exposure to outcome. Leave-one-out sensitivity analyses were also performed to evaluate the robustness of the results.
8	**Sensitivity analyses and additional analyses**		Describe any sensitivity analyses or additional analyses performed (e.g. comparison of effect estimates from different approaches, independent replication, bias analytic techniques, validation of instruments, simulations)	8	The estimators from other four methods were included as robust sensitivity analyses. All effect estimates are reported as β coefficients representing the SD change in the outcome per SD increase in the exposure.

9	**Software and pre-registration**				
		a)	Name statistical software and package(s), including version and settings used	9	All MR analyses were performed using the TwoSampleMR package (version 0.6.2) in R software (version 4.4.0)(16).
		b)	State whether the study protocol and details were pre-registered (as well as when and where)		N/A
	**RESULTS**				
10	**Descriptive data**				
		a)	Report the numbers of individuals at each stage of included studies and reasons for exclusion. Consider use of a flow diagram		N/A
		b)	Report summary statistics for phenotypic exposure(s), outcome(s), and other relevant variables (e.g. means, SDs, proportions)		N/A
		c)	If the data sources include meta-analyses of previous studies, provide the assessments of heterogeneity across these studies		N/A
		d)	For two-sample MR:<br>i. Provide justification of the similarity of the genetic variant-exposure associations between the exposure and outcome samples<br>ii. Provide information on the number of individuals who overlap between the exposure and outcome studies		Both the Pan-UKB and FinnGen R12 datasets included participants of European ancestry. Although recruited from different populations, the genetic architecture is expected to be highly comparable between these cohorts. Therefore, the genetic variant-exposure associations derived from the Pan-UKB can be reasonably assumed to apply to the FinnGen population. No substantial sample overlap is expected between the Pan-UKB and FinnGen datasets, as these cohorts were recruited independently from distinct geographic regions.
11	**Main results**				
		a)	Report the associations between genetic variant and exposure, and between genetic variant and outcome, preferably on an interpretable scale	9	The strength of these instrumental variables was confirmed by F-statistics ranging from 29.8 to 1816.0 for vitamin D and from 29.8 to 948.4 for height, well above the conventional threshold of 10, minimizing the risk of weak instrument bias.
		b)	Report MR estimates of the relationship between exposure and outcome, and the measures of uncertainty from the MR analysis, on an interpretable scale, such as odds ratio or relative risk per SD difference	9-10	The MR analysis indicated a significant positive causal effect of vitamin D concentrations on adult height. As shown in Table 1, the primary IVW method estimated that each one SD increase in genetically predicted 25OHD concentration was

					associated with a 0.046 SD increase in adult height ($p = 1.53 \times 10^{-5}$).<br>In the reverse direction, we found no evidence to support a causal effect of adult height on circulating vitamin D levels. As shown in Table 1, the IVW estimate was small and non-significant (β = 0.008, p = 0.343).
		c)	If relevant, consider translating estimates of relative risk into absolute risk for a meaningful time period		N/A
		d)	Consider plots to visualize results (e.g. forest plot, scatterplot of associations between genetic variants and outcome versus between genetic variants and exposure)	9-10	Figure 2
12	**Assessment of assumptions**				
		a)	Report the assessment of the validity of the assumptions	9-10	The strength of these instrumental variables was confirmed by F-statistics ranging from 29.8 to 1816.0 for vitamin D and from 29.8 to 948.4 for height, well above the conventional threshold of 10, minimizing the risk of weak instrument bias.<br>The Steiger directionality test confirmed the direction of effect from vitamin D to height (p reported as 0 due to computational lower bound).<br>No directional pleiotropy was detected (MR-Egger intercept p = 0.432), and the Steiger test again validated the causal orientation (p reported as 0 due to computational lower bound).
		b)	Report any additional statistics (e.g., assessments of heterogeneity across genetic variants, such as I^2, Q statistic or E-value)	10	Moderate heterogeneity was observed across instruments (I^2 = 59.5%).<br>While there was moderate heterogeneity among instruments (I^2 = 40.2%), the findings across sensitivity analyses converged on the absence of a reverse causal effect.
13	**Sensitivity analyses and additional analyses**				
		a)	Report any sensitivity analyses to assess the robustness of the main results to violations of the assumptions	9-10	While the MR-Egger point estimate was positive, it did not reach statistical significance (p = 0.148), and the MR-Egger intercept test revealed no evidence of directional pleiotropy (p = 0.094).

					No directional pleiotropy was detected (MR-Egger intercept p = 0.432), and the Steiger test again validated the causal orientation (p reported as 0 due to computational lower bound).
		b)	Report results from other sensitivity analyses or additional analyses	9-10	Supporting this result, both the weighted median ($\beta = 0.031$, $p = 2.74 \times 10^{-3}$) and weighted mode ($\beta = 0.034$, $p = 2.10 \times 10^{-4}$) methods yielded consistent effect estimates.<br>Other MR methods, including MR-Egger (p = 0.262) and weighted median (p = 0.160), were consistent in direction and statistical insignificance.
		c)	Report any assessment of direction of causal relationship (e.g., bidirectional MR)	9-10	Yes
		d)	When relevant, report and compare with estimates from non-MR analyses		N/A
		e)	Consider additional plots to visualize results (e.g., leave-one-out analyses)	9-10	Figure 2
	**DISCUSSION**				
14	**Key results**		Summarize key results with reference to study objectives	10	In this bidirectional two-sample MR study, we found evidence supporting a causal relationship between genetically predicted circulating vitamin D concentrations and adult height, while no causal effect of genetically predicted height on vitamin D levels was observed. The consistency across multiple MR estimators, together with the absence of substantial horizontal pleiotropy, strengthens the robustness of these findings.
15	**Limitations**		Discuss limitations of the study, taking into account the validity of the IV assumptions, other sources of potential bias, and imprecision. Discuss both direction and magnitude of any potential bias and any efforts to address them	13	Nonetheless, certain limitations warrant consideration. First, although no substantial pleiotropy was detected, residual pleiotropy cannot be completely excluded. Second, the use of adult vitamin D instruments to proxy developmental exposures may underestimate the true causal effect during specific growth windows. Third, height was inverse-rank normalized in the FinnGen cohort, complicating the translation of effect sizes into centimeters. Finally, our findings may not generalize to non-European populations, where genetic determinants of vitamin D status and environmental exposures differ.
16	**Interpretation**				

a)	Meaning: Give a cautious overall interpretation of results in the context of their limitations and in comparison with other studies	11-12	Our findings are consistent with, yet extend beyond, the evidence from RCTs. Several large-scale RCTs have demonstrated that although vitamin D supplementation significantly raises circulating 25OHD levels, it does not substantially enhance linear growth in children and adolescents(8, 9, 12). For example, phase 3 trials conducted in Mongolian (14,000 IU/week for 3 years) and South African children (10,000 IU/week for 3 years) failed to show significant growth benefits after long-term supplementation, in contrast to an earlier phase 2 trial in Mongolian children, where daily supplementation with 800 IU of vitamin D_3 for six months led to a modest but statistically significant increase in height among those with very low baseline serum vitamin D levels(9, 12, 30). Meta-analyses have further corroborated these negative findings(13, 14). However, it is important to recognize that these RCTs may have been underpowered to detect modest effects due to small expected effect sizes, relatively short follow-up durations, and limited sample sizes. By contrast, MR analyses leverage genetic variants as lifelong proxies for exposure, providing greater statistical power to detect small but genuine causal effects. The positive association observed in our study suggests that vitamin D does contribute to linear growth, but the magnitude of its influence is too subtle to be readily captured within the constraints of conventional RCT designs.
b)	Mechanism: Discuss underlying biological mechanisms that could drive a potential causal relationship between the investigated exposure and the outcome, and whether the gene-environment equivalence assumption is reasonable. Use causal language carefully, clarifying that IV estimates may provide causal effects only under certain assumptions	12	From a biological perspective, vitamin D promotes skeletal development by facilitating calcium and phosphate absorption, stimulating chondrocyte proliferation within the growth plate, and modulating insulin-like growth factor-1 (IGF-1) signaling(1, 31, 32). Severe deficiency leads to rickets and growth failure, as observed in VDR-knockout models and clinical settings(33, 34). Recent two-sample Mendelian randomization analyses have further supported a bidirectional causal relationship between circulating 25OHD and IGF-1 levels(35).
c)	Clinical relevance: Discuss whether the results have clinical or public policy relevance, and to what extent they inform effect sizes of possible interventions	12	Despite these mechanistic insights, our estimates suggest that in vitamin D-replete populations, each 1-SD increase in 25OHD is associated with only a

				0.046 SD increase in height, corresponding to a clinically modest impact. From a public health perspective, our results reinforce the importance of maintaining adequate vitamin D status for skeletal health but indicate that supplementation alone is unlikely to produce substantial height gains in otherwise healthy individuals. Therefore, strategies should prioritize preventing overt vitamin D deficiency, particularly during critical periods of growth, rather than expecting notable gains in stature through universal supplementation.
17	**Generalizability**	Discuss the generalizability of the study results (a) to other populations, (b) across other exposure periods/timings, and (c) across other levels of exposure	13	our findings may not generalize to non-European populations, where genetic determinants of vitamin D status and environmental exposures differ.
	**OTHER INFORMATION**			
18	**Funding**	Describe sources of funding and the role of funders in the present study and, if applicable, sources of funding for the databases and original study or studies on which the present study is based		N/A
19	**Data and data sharing**	Provide the data used to perform all analyses or report where and how the data can be accessed, and reference these sources in the article. Provide the statistical code needed to reproduce the results in the article, or report whether the code is publicly accessible and if so, where	2	Data Availability Statement<br>All data used in this study are publicly available. Summary-level genetic association data for circulating 25-hydroxyvitamin D concentrations were obtained from the Pan-UK Biobank resource (http://www.nealelab.is/uk-biobank/). Adult height data were sourced from the FinnGen consortium (https://www.finngen.fi/en). Additional information can be obtained from the corresponding author upon reasonable request.
20	**Conflicts of Interest**	All authors should declare all potential conflicts of interest	2	Conflicts of Interest<br>The authors declare that they have no commercial or financial relationships that could be construed as potential conflicts of interest in relation to this study.

This checklist is copyrighted by the Equator Network under the Creative Commons Attribution 3.0 Unported (CC BY 3.0) license.

1. Skrivankova VW, Richmond RC, Woolf BAR, Yarmolinsky J, Davies NM, Swanson SA, et al. Strengthening the Reporting of Observational Studies in Epidemiology using Mendelian Randomization (STROBE-MR) Statement. JAMA. 2021;under review.

2. Skrivankova VW, Richmond RC, Woolf BAR, Davies NM, Swanson SA, VanderWeele TJ, et al. Strengthening the Reporting of Observational Studies in

Epidemiology using Mendelian Randomisation (STROBE-MR): Explanation and Elaboration. BMJ. 2021;375:n2233.
