# Supplementary material for: Causal relationship between vitamin D and adult height: A bidirectional Mendelian randomization study
Source: Medicine (Baltimore). 2025 Aug 29;104(35):e44123. doi: 10.1097/MD.0000000000044123 (PMC12401308; doi:10.1097/MD.0000000000044123)
Supplement: Supplementary file 2 [file medi-104-e44123-s002.docx]

Table S1. Data source of genome-wide association studies included in the Mendelian randomization analysis

| Phenotype | Sources | Sample size | SNPs | Population |
| --- | --- | --- | --- | --- |
| 25-hydroxyvitamin D | Pan-UKB | 383,324 | 28,987,534 | European Ancestry |
| Height* | Finngen R12 | 364,629 | 21,324,955 | European Ancestry |

* Data is inverse-rank normalized.
